# *In vivo* histone H1 migration from necrotic to viable tissue

**DOI:** 10.18632/oncotarget.15181

**Published:** 2017-02-07

**Authors:** Keith A. Luhrs, Desmond Pink, Wendy Schulte, Andries Zijlstra, John D. Lewis, Missag H. Parseghian

**Affiliations:** ^1^ Allergan Inc., Irvine, CA, USA; ^2^ Innovascreen Inc., Halifax, NS, Canada; ^3^ Vanderbilt University Medical Center, Nashville, TN, USA; ^4^ University of Alberta, Edmonton, AB, Canada; ^5^ Rubicon Biotechnology, Lake Forest, CA, USA; ^6^ Peregrine Pharmaceuticals Inc., Tustin, CA, USA

**Keywords:** tumor microenvironment, drug delivery, histone H1, protein trafficking, necrosis

## Abstract

Necrosis is induced by ischemic conditions within the core of many solid tumors. Using fluorescent fusion proteins, we provide *in vivo* evidence of histone trafficking among cancer cells in implanted tumors. In particular, the most abundant H1 isoform (H1.2) was found to be transported from necrotic tumor cells into surrounding viable cells where histones are selectively taken up by energy-dependent endocytosis. We propose that intercellular histone trafficking could function as a target for drug delivery. This concept was validated using an anti-histone antibody that was co-internalized with histones from dead cells into viable ones surrounding the necrotic regions of a tumor, where some of the most chemoresistant cells reside. These findings demonstrate that cellular translocation of conjugated drugs using anti-histone antibodies is a promising strategy for targeted drug delivery to chemoresistant tumors.

## INTRODUCTION

The classical role of histones as organizers of genomic DNA has evolved in recent decades with the elucidation of their function in encoding epigenetic information. Their roles have been further expanded by work that implicates them in sophisticated cell signaling pathways related to diverse processes that include innate immunity (as reviewed in [1]). For instance, release of a specific histone H1 subtype (H1.2) from the nucleus into the cytoplasm, the result of a DNA double-strand break, triggers the liberation of cytochrome C from the mitochondria, resulting in apoptosis [2]. Histones have also been found residing in the plasma membrane and functioning as thyroglobulin receptors on macrophages [3] and bacterial CpG oligodeoxynucleotide receptors on teleost natural killer cells [4]. As an expanding body of literature describing the cell penetrating properties of histones has been accumulating, some suggest that the observations are a fixation artifact and that proper experimentation requires the use of unfixed, living cells [5]. Interestingly, several studies have demonstrated core histone [6, 7] and linker histone [8] translocations across liposomes and cell membranes *in vitro*. In intact cells, core histones exhibit different degrees of cellular internalization independent of ATP-driven endocytosis (clathrin or caveoloe-mediated) or pinocytosis [6]. Moreover, studies with fluorescently-labeled H1 have revealed that translocation is dependent on the presence of phosphatidyl serine (PS), which causes a pronounced increase in H1 secondary structure and its aggregation on the membrane surface, followed by uptake either into liposomes or cultured leukemic T-cells [8]. Despite mounting evidence for histone translocation *in vitro*, it has remained unclear whether this is a physiologically relevant phenomenon.

Regions of significant necrosis, such as the core or perinecrotic regions of a solid tumor, are difficult to treat with existing therapies due to poor targeting, uptake and chemoresistance. We have considered the intriguing possibility that histones released from dead cells and translocated into surrounding living cells *in vivo* could provide a viable target for delivery of antibody-drug conjugates into a tumor's hypoxic core and adjacent cells. *In vitro* evidence supporting such a mechanism is seen in tumor cell cultures treated with low doses of dexamethasone and vincristine, causing partial cell death (∼25%) and a 10- to 12-fold increase in extracellular nucleosomes (NS). This, in turn, results in a 50-fold increase in the binding of an anti-NS (MoAb 2C5) to the surface of the surviving tumor cells [9]. ^125^I-labeled NS have been observed translocating into cultured fibroblast cells and their internalization rate increases when bound by anti-histone or anti-DNA antibodies [10]. In light of these observations, we have conducted further investigations into histone H1 migration *in vitro* and *in vivo*. We provide evidence demonstrating histone migration and translocation of the most abundant H1 isoform (H1.2 or H1^S^-1) [11] can occur *in vivo*, and that translocating histones can be targeted using a well-characterized anti-histone/DNA antibody.

## RESULTS

### Characterization of a novel histone/DNA targeting antibody

**Figure 1:**
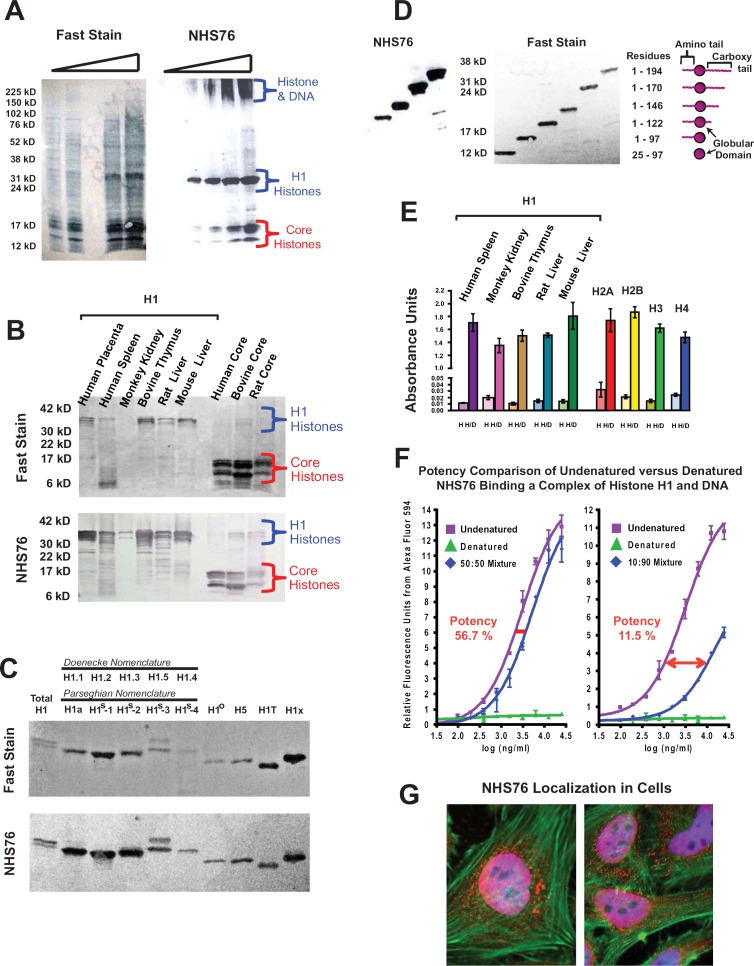
NHS76 specificity studies **A**. Increasing quantities of whole cell extracts were probed on western blots to verify NHS76 specificity (*left panel*: a general protein stain known as “Fast Stain” was used to reveal all of the proteins on the blot; *right panel*: immunoblot with NHS76). Bands detected correspond to the apparent molecular weights for H1 and core histones characteristically seen for these highly basic proteins, their degradation fragments and an aggregation of histones bound to DNA that barely penetrates the top of the gel. **B**. NHS76 detects a common motif present in all five families and across mammalian species despite denaturation of the proteins in the SDS-PAGE (*upper panel*: Fast Stain; *lower panel*: NHS76). **C**. NHS76 was tested against a panel of nine known H1 isoforms (excluding gamete specific H1Foo and HILS1). The common H1 nomenclature systems are listed (13, 40). The epitope is detected on all nine by western blotting (upper panel: Fast Stain; lower panel: NHS76). **D**. Deletion mutants of a single H1 subtype were used to localize the epitope on the histone to the C-terminal tail proximal to the globular domain (*left panel*: NHS76; *middle panel*: Fast Stain; *right panel*: Schematic of bands and corresponding H1 fragments). **E**. The epitope is also detected in its native conformation when probing an ELISA plate containing H1 histones from different species and all 4 types of core histones (H columns). The epitope is accentuated with the binding of histones to DNA (H/D columns). Results from the negative control, consisting of wells probed only with the secondary antibody, were subtracted from the corresponding wells probed with NHS76 as detailed in the Experimental Procedures. **F**. Denaturation of the human antibody, NHS76, disrupts its binding to DNA (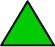, 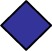) demonstrating interaction of undenatured antibody to the H1/DNA complex (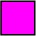) is not the result of a non-specific attraction. **G**. *In vitro* staining of fixed cells with NHS76. Antibodies were localized to the histones in the nucleus and cytoplasm using Alexa-594 conjugated goat anti-human (red). DNA was co-localized to the nucleus with DAPI (blue). Merged images of red nuclei and blue DAPI gives the nuclei a lavender appearance. The cytoskeletal actin was illuminated with Alexa-488 conjugated phalloidin (green).

To study the possibility that histones released from dead cells could provide a viable target for delivery of antibody-drug conjugates into surrounding living cells, we chose a human anti-histone antibody (NHS76) that targets an epitope which is conformationally stable upon histone binding to DNA. NHS76 was originally generated to target the immunoaccessible H1 histones, yet it was found to cross-react with all 4 types of core histones as well. To rule out NHS76 cross-reactivity with non-histone proteins, it was tested against whole cell extracts using western blots and found only to bind histones (Figure 1A). NHS76 detects the same epitope across multiple mammalian species under the denaturing conditions of an SDS-PAGE and it is sensitive enough to detect minor amounts of degradation in the purified H1 samples (Figure 1B). The epitope recognized by the NHS76 antibody is present across nine of the known H1 subtypes [12], allowing for a single subtype to be representative in further characterization studies (Figure 1C). The epitope was identified using deletion mutants of subtype H1° on a western blot, revealing its presence on the C-terminal tail (Figure 1D). The increased signal strength with the lengthier molecules suggests the epitope may be a repeated motif across the tail sequence, which would place it around the S/TPKK or KPKAA repeat sequences [13]. ELISAs reveal that the epitope is detected on histone proteins in a native conformation, however, the structure is 100-fold more accessible when the histones bind DNA (compare the signal obtained with each histone [H] to its own histone-DNA complex [H/D] in Figure 1E). To counter suggestions that increased binding is due to a non-specific electrostatic interaction between the relatively basic human IgG1 constant region on NHS76 (pI=8.5) and the acidic DNA phosphate backbone, we denatured NHS76 at 90°C for 15 minutes in Tris Buffered Saline (TBS), then cooled it on ice prior to mixing it with its undenatured form at 50:50 and 1:10 dilutions (Figure 1F). Denatured and undenatured NHS76 were then tested in a dose dependent manner against an H1/DNA complex (1:5 weight ratio) to measure the binding potency of the antibodies. An antibody constant region that binds DNA non-specifically should not be affected by denaturation; however, the results show that the binding potency of the denatured NHS76 was nil and that the 50:50 and 1:10 dilutions resulted in 56.7% and 11.5% potencies commensurate with the amount of denatured antibody in the cocktails. Finally, functional NHS76 detects histones in the nucleus and the cytoplasm of cells *in vitro* (Figure 1G). Co-staining with DAPI, a DNA specific dye, illustrates the presence of NHS76 at the cell nucleus. NHS76 can also clearly detect histones in the cytoplasm that are ready for transport into the cell nucleus [14].

The lengthier the DNA molecule, the greater number of histones that can bind, therefore, affinity was studied by creating a 1 histone : 1 DNA structure. The creation of a cruciform structure using 4 distinct strands of DNA has been described previously [15] and was modified by the placement of a biotin molecule at the 5′ end of one strand. A single molecule of subtype H1.2, will bind the cruciform 4-way DNA structure and provide a well-defined entity for binding studies [16]. Biolayer interferometry [17], a label-free kinetic method, was used to monitor assembly of complexes produced on streptavidin coated biosensors (see Supplementary Figure 1 for a full description). NHS76 antibody was found to bind DNA alone, histone H1 alone, as well as the DNA/H1 complex. Although direct calculation of affinity is complicated by the complex nature of the target, data suggests the interaction to be in the mid-nanomolar to micromolar range, which is relatively weak. Based on observations first described in tumors [18], a relatively weaker affinity antibody can be advantageous in allowing deeper penetration of the antibody into the tumor core.

### Cellular uptake of histone H1 is mediated by energy-dependent endocytosis

**Figure 2:**
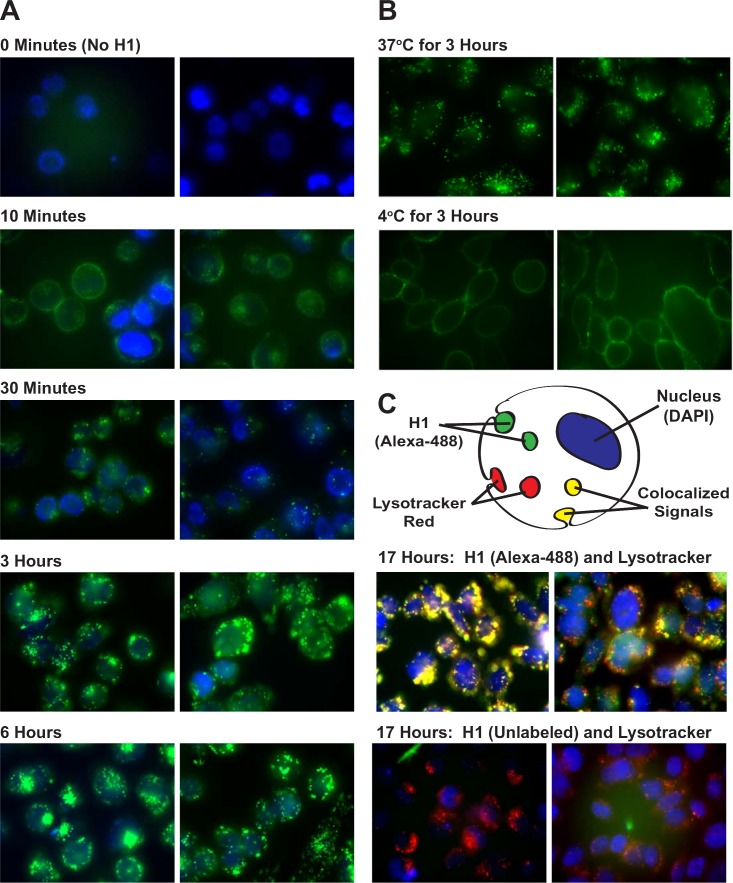
Live Cell Imaging of H1 Uptake by Endocytosis **A**. Live, unfixed, non-permeabilized CHO cells were incubated with Alexa-488 labeled H1 (5 μg/ml) at 37°C and visually tracked over 6 hours. Before imaging, cells were incubated with DAPI at a high enough concentration (10 μg/ml) to stain nuclei within intact cells. Endosomal vesicles were observed in all 4 repetitions of this study. **B**. CHO cells were incubated for 3 hours with Alexa-488 H1 (10 μg/ml) at 37°C or 4°C. Vesicle formation can be seen in the 37°C cells while the 4°C cells exhibit a dim peripheral staining. **C**. Cells incubated with Alexa-488 or unlabeled H1 (10 μg/ml) at 37°C for 17 hours were further stained, during the last 30 minutes prior to microscopy, with 50 nM Lysotracker Red and 100 μg/ml Hoechst 33258, a cell permeable DNA stain.

Previous studies have not clearly demonstrated whether histone uptake uses energy-driven endocytosis or a novel translocation mechanism [6, 8]. Part of the confusion is related to the fact that several previous studies investigated histone uptake within 1 hour of exposure, despite evidence suggesting it takes 16 hours before serum DNAse I and plasmin begin degrading nucleohistones in necrotic tissues [19]. To determine the cellular uptake mechanism for histone H1, proteins were labeled with Alexa-488, incubated with live cells and visualized using fluorescence microscopy over 17 hours (Figure 2A). CHO cells incubated with Alexa-488 labeled H1 (green) showed visible intracellular vesicle staining within 30 minutes and significant accumulation of signal over 17 hours (Figures 2A and 2C). Extracellular signals were quenched using 0.25 mg/mL crystal violet and 0.001% Triton X-100; concentrations that did not cause cell permeabilization (data not shown). Very little H1 uptake was observed when cells were incubated at 4°C compared to 37°C (Figure 2B). After 17 hours of incubation at 37°C, significant colocalization was seen between histone H1 and the acidic endosomal compartment, visualized using Lysotracker Red DND-99 (Figure 2C, upper panel). Vesicle formation due to the Alexa-488 label was ruled out when unlabeled H1 also stimulated endosomal vesicles as seen with Lysotracker Red (Figure 2C, lower panel). Similar results were obtained with N87 gastric carcinoma cells (Supplementary Figure 2A).

**Figure 3:**
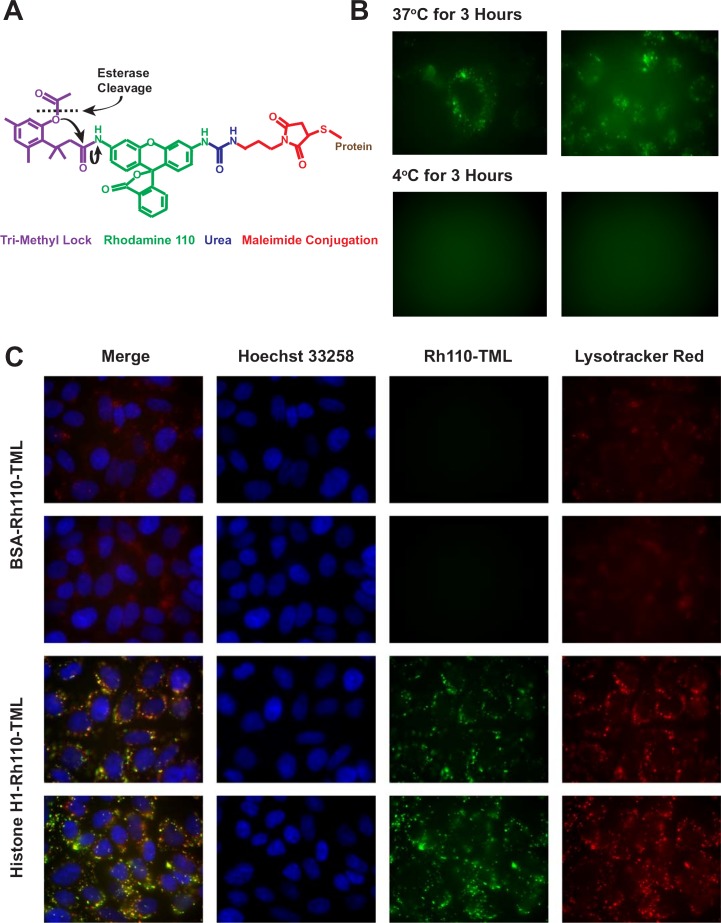
Confirmation of uptake using a latent fluorophore **A**. Upon cellular internalization, ester hydrolysis of the Rh110-TML releases the trimethyl lock moiety through nucleophilic reactions (black arrows) and unmasks rhodamine fluorescence. **B**. CHO cells were incubated for 3 hours with H1-Rh110-TML (10 μg/ml) at 37°C or 4°C. Vesicle formation can be seen in the 37°C cells. The 4°C cells lacked any discernible staining so a focused image was not obtained. **C**. Rh110-TML-labeled BSA or histone H1 (both 10 μg/ml) were incubated with CHO cells for 17 h. During the final 30 min, 50 nM LysoTracker Red and 100 μg/ml Hoechst 33258 were added. Images were taken of live, unfixed cells. Endosomal vesicles were observed with H1-Rh110-TML in all 3 repetitions of this study. Exposure times and image processing were identical for each image.

To confirm the uptake path of histone H1 into acidic endosomes without the complication of signal quenching with crystal violet and Triton X-100, the “latent fluorophore” maleimidourea-rhodamine-110-trimethyl lock (Rh110-TML) was utilized [20]. A rhodamine derivative that can be conjugated to proteins with a maleimide linkage, Rh110-TML is a quenched fluorophore that is unmasked upon esterase cleavage, as occurs in endosomes and the cytosol (Figure 3A). Incubation of CHO cells with H1-Rh110-TML at 37°C and 4°C confirmed that, while significant endosomal uptake is seen at 37°C, no uptake is seen at 4°C (Figure 3B). Co-localization of the H1 to acidic endosomes was reconfirmed using Lysotracker Red (Figure 3C). Besides indicating that H1 is internalized via an endocytic pathway, the lack of endosomal vesicles when using a Bovine Serum Albumin (BSA-Rh110-TML) control suggests that the presence of H1 may stimulate endocytosis. Similar results were obtained with N87 gastric carcinoma cells (Supplementary Figure 2B). To quantitate the relative difference in histone uptake to BSA, we chose two cell lines that adhere to the bottom of a 96-well plate, allowing for the reading of fluorescent signal from the internalized Rh110-TML labelled proteins (Supplementary Figure 3A).

### Histone H1.2 migration and translocation occurs *in vivo*

**Figure 4:**
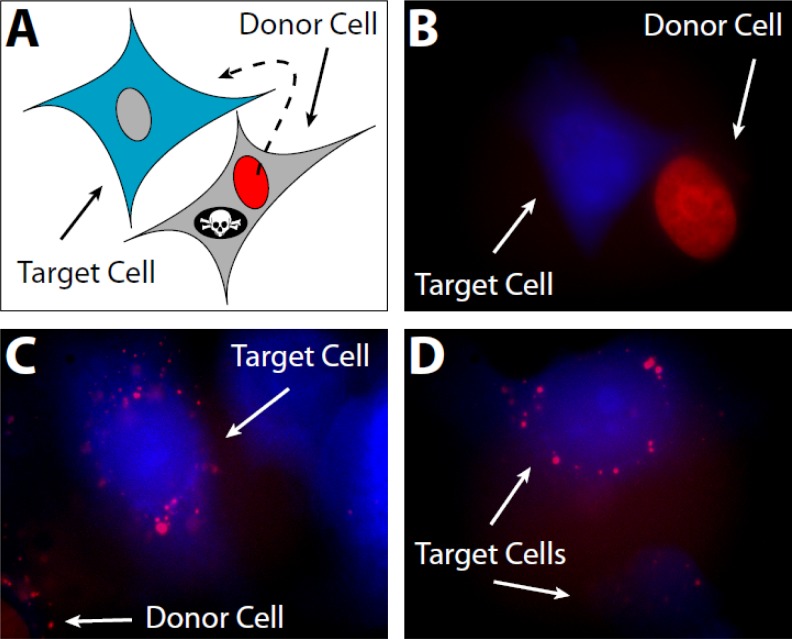
I*n vitro* histone migration from necrotic cells to viable ones **A**. To model the perinecrotic region of a tumor, HT1080 donor cells expressing red-fluorescent histone (mCherry-H1.2) and a suicide gene (HSV1-tk) are plated alongside target cells labeled with 100 μM CMHC blue fluorescent tracer. CMHC penetrates viable cells and is converted into a cell-impermeant form; hence, it can be passed to daughter cells through several generations but does not diffuse into surrounding donor cells. **B**. Viable donor and target cells before selective induction of donor cell death. mCherry-H1.2 localizes in the nucleus, whereas CMHC is in the whole cell. **C**. Histones are internalized by target cells in the vicinity of a dying donor cell after induction of donor cell death with 50μM ganciclovir. Red vesicles in blue cells were lacking in tissue cultures not incubated with ganciclovir (data not shown). **D**. More viable target cells possessing histones from necrotic donor cells.

To test the concept of H1 migration from necrotic cells to viable ones in the tumor microenvironment, we created a model *in vitro* system using human fibrosarcoma cells (HT1080). Donor cells were created by transfecting with a vector encoding the most abundant isoform of H1 (H1.2 *Doenecke nomenclature*; H1^S^-1 *Parseghian nomenclature*) fused to mCherry [21] and encoding the HSV1-tk gene to allow selective induction of cell death upon exposure to ganciclovir. Untransfected target cells were labeled with Cell Tracker Blue CMHC (Invitrogen) (Figure 4). In this system, red-fluorescent H1-mCherry/HSV1-tk expressing donor cells and viable blue target cells can be positively identified in co-culture (Figures 4A and 4B). Upon induction of cell death with ganciclovir, mCherry H1.2 from dead and dying donor cells was observed being taken up by blue-fluorescent target cells (Figures 4C and 4D). For the subsequent *in vivo* experiments, three stable transfectants were then created: 1) “inducible death” donor cells expressing mCherry-H1.2 and HSV1-tk, 2) inducible death control donor cells, referred to as “mCherry-Empty” expressing only mCherry and HSV1-tk, and 3) target cells expressing Green Fluorescent Protein (GFP). A panel of stable transfectants was created for each of the following tumor lines: HT1080, MDA-MB-231, and MDA-MB-435.

**Figure 5:**
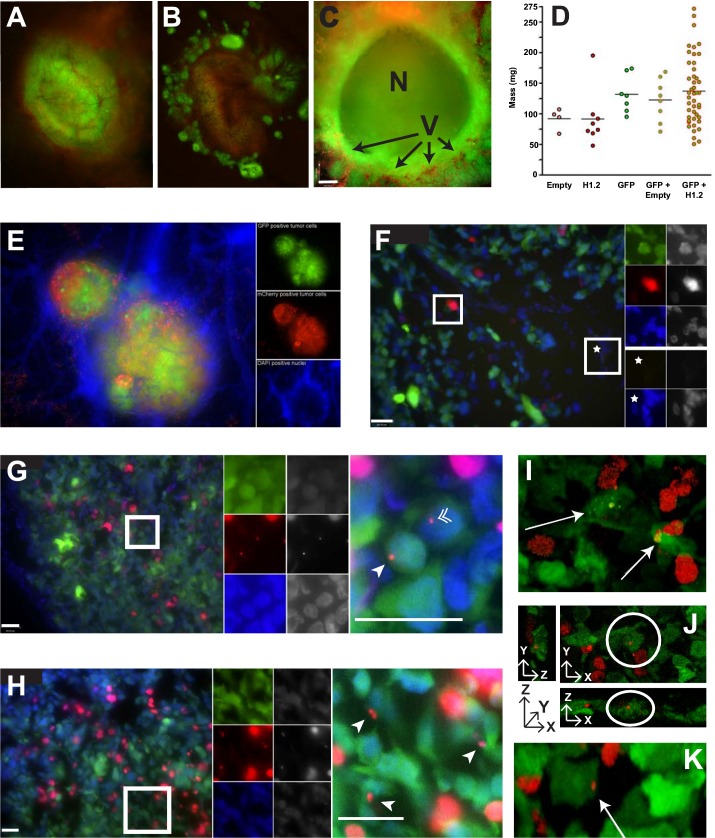
*In vivo* histone migration from necrotic cells to viable ones **A**. Mixtures of mCherry-H1.2 donor cells and GFP target cells exhibit green colored tumors with punctate red spots indicative of red nuclei from the MDA-MB-435 donor cells. **B**. Mixtures of mCherry-Empty donors with GFP targets produced a brownish tumor due to the diffuse distribution of mCherry and GFP in the cytoplasm of their respective cells. **C**. Section of a spheroid mass formed by GFP-expressing MDA-MB-435 cells reveals a significant amount of necrosis (N) within these tumors grown on the CAM. The chicken vasculature (V) was visualized with Alexa-Fluor 750 labeled *cowpea mosaic virus (CPMV-AF750 in red; (41))* and reveals penetration of blood vessels into the tumor mass (bar = 260 μm). **D**. Distribution of masses for tumors consisting of mCherry-Empty expressing cells (Empty), mCherry-H1.2 expressing cells (H1.2), GFP expressing cells (GFP), 1:1 mixtures of GFP and mCherry-Empty, or 1:1 mixtures of GFP and mCherry-H1.2. **E**. Merged (*left panel*) and fluorophore-specific images (GFP, mCherry and DAPI *from top to bottom on right panels*) of MDA-MB-435 tumor section stained with DAPI (with ProLong^®^ Gold antifade) to reveal all intact nuclei including non-tumor cells. Merged images of red mCherry-H1.2 nuclei and blue DAPI gives those nuclei a lavender appearance. **F**. Wide field image from 1:1 mix of mCherry-Empty:GFP tumor with magnification of two regions (bar = 100 μm). *Left panel*, merged wide field image; *top right panels*, magnification of boxed region populated with viable tumor cells; *bottom right panels*, magnification of region without tumor. Fluorophore-specific images (*top*: GFP, mCherry and DAPI; *bottom*: GFP and DAPI) on the right and far right panels are false color and gray-scale images, respectively. **G**. Wide field image from 1:1 mix of mCherry-H1.2 : GFP tumor (bars = 100 μm). *Left panel*, merged wide field image; *middle panels*, fluorophore-specific images of boxed region; *right panel*, magnification of boxed region revealing mCherry-H1.2 signal in GFP cells, including the cytoplasm (arrowhead) and nuclei (double arrowhead). **H**. Another example. Same legend as (G). **I**. Confocal image of 10μm tumor section. mCherry-H1.2 signal is seen in the cytoplasm of GFP cells (arrows). **J**. Confocal image of another 10μm tumor section viewed from three angles confirms mCherry-H1.2 signal in cytoplasm of GFP cells (circled) [XY (*top right panel*) XZ (*bottom*) and YZ (*left*) views]. **K**. Three-dimensional confocal image of another 10 μm tumor section revealing mCherry-H1.2 signal in the GFP cytoplasm (arrow).

To test whether H1 migration occurs *in vivo*, human tumor xenografts were established on the chorioallantoic membrane (CAM) of *ex ovo* chicken embryos [22]. Easily accessible for experimental manipulation, the CAM is a highly vascularized extraembryonic membrane connected to the chick embryo through a continuous circulatory system. Implantation of xenograft human tumors on the membrane of *ex ovo* embryos permits real-time visualization of intratumoral dynamics [22]. Tumors for our *in vivo* system were generated from a 1:1 mixture of cells expressing either mCherry-H1.2 or un-fused mCherry with cells expressing cytoplasmic GFP (Figures 5A and 5B, respectively). Tumors were heterogeneous with apparent spheroid masses, and areas of probable necrosis were seen (Figure 5C). Tumors consisting of a single cell type or as mixtures were compared by weight to determine if expression of any particular transfectant had an impact on tumor growth. No significant difference in tumor mass was observed (Figure 5D). To locate non-tumor cells *in vivo*, particularly endothelial cells, DAPI was intravenously injected near the tumors prior to imaging (Figure 5E).

Tumors with visible areas of necrosis were harvested, fixed, frozen and sectioned for microscopy followed by DAPI staining to visualize all nuclei. In the tumors generated from a 1:1 mix of mCherry-Empty and GFP cells, the unfused mCherry expressing cells appeared intact and viable with the fluorescence restricted only to these cells (Figure 5F). In tumors created from a 1:1 mix of mCherry-H1.2 and GFP cells, distinct areas of mCherry-H1.2 signal were observed within the cells expressing GFP (Figures 5G-5K). Fluorescent mCherry-H1.2 could be seen in both the cytoplasm and in the nuclei of the GFP-expressing target cells (Figure 5G). The presence of mCherry-H1.2 on the surface of multiple GFP cells suggests that the histones are widely dispersed when necrosis leads to chromatin release (Figure 5H). The presence of mCherry-H1.2 in the interior of GFP cells was confirmed by 3D confocal analyses in Figure 5I, 5J, and 5K.

### Translocating histones can be targeted *in vivo* using NHS76

**Figure 6:**
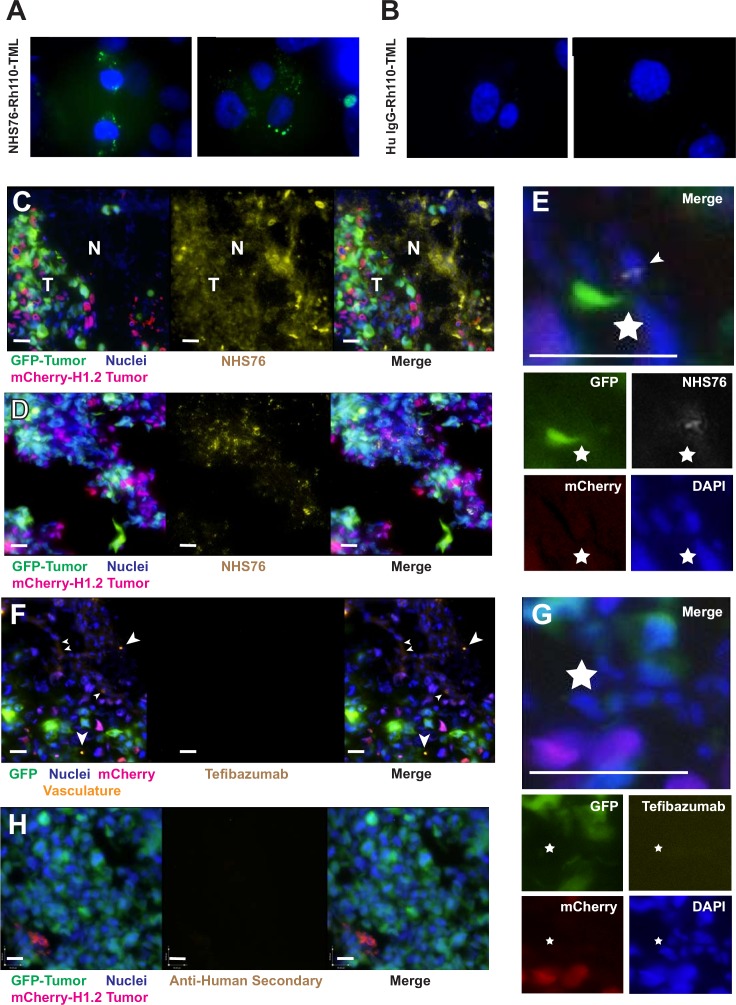
*In vivo* antibody migration from necrotic cells to viable ones **A**. HT1080 cells were incubated for 6 hours with NHS76-Rh110-TML (∼933 nM) at 37°C in a 5% CO_2_ incubator. Vesicle formation correlates to ester hydrolysis of the Rh110-TML and unmasking of fluorescence upon cellular internalization. **B**. No signal was discernible using a polyclonal mix of human IgG_1_ / lambda antibodies (∼933 nM). DAPI staining (10 μg/ml) provided dim staining of the nuclei within viable cells and bright illumination within dead ones, hence, allowing us to differentiate the two and focus on cells with dimly stained nuclei. Exposure times and image processing were identical for both figures. **C**. Merged images of tumor cells (*left panel*: GFP, mCherry and DAPI) and NHS76 antibody (*middle panel*) reveals distribution of NHS76 in viable tumor (T) as well as necrotic regions (N) of cellular debris (*right panel*). NHS76 was detected using a 2-step approach of rabbit anti-human IgG (5 μg/mL) followed by goat anti-rabbit conjugated to Alexa-750 (1 μg/mL). Before imaging, cells were incubated with DAPI (with ProLong^®^ Gold antifade) to reveal all intact nuclei including non-tumor cells. Merged images of red mCherry-H1.2 nuclei and blue DAPI gives those nuclei a lavender appearance (bars = 90 μm). **D**. Another example of NHS76 distribution among viable tumor cells. Same legend as (C). **E**. NHS76 internalization (arrowhead) is shown using a goat anti-human secondary conjugated to a quantum dot (655 nm) in the merged image (*upper panel*; bar = 100 μm; star used for image alignment) generated from the fluorophore-specific images for GFP, mCherry, DAPI and NHS76 (*lower panels*). **F**. Merged images of tumor cells and vasculature (*left panel*: GFP, mCherry, DAPI and LCA [arrowheads]) and the control antibody tefibazumab (*middle panel*) show no detectable distribution within the tumor microenvironment (*right panel*). Secondary antibody and DAPI details same as (C); bars = 90 μm. **G**. Internalization is not seen with tefibazumab either in the merged image (*upper panel*; bar = 100 μm; star used for image alignment) or the fluorophore-specific images (*lower panels*). **H**. Lack of secondary antibody crossreactivity was verified by injecting PBS rather than antibody and subsequently tissue staining with rabbit anti-human IgG followed by goat anti-rabbit conjugated to Alexa-750 (bars = 90 μm).

Our observation that histones are released from dead cells and translocated into surrounding living cells *in vivo* suggests that they could provide a viable target for delivery of antibody-drug conjugates into a tumor's hypoxic core. To demonstrate co-internalization of anti-histone antibodies with their antigenic targets into cells, the latent fluorophore Rh110-TML was conjugated to NHS76. Internalization of labeled NHS76 is shown in Figure 6A. Since the NHS76 antibody has an IgG_1_ constant region and a lambda light chain, a polyclonal mix of human IgG_1_/lambda was labeled with Rh110-TML as a control for internalization of non-specific antibody, which did not occur (Figure 6B). For most of these experiments, the antibodies were labeled with Rh110-TML and the histones unlabeled, with the exception of the positive control, in which H1-Rh110-TML was used to verify the internalization process (data not shown). Anti-histone internalization was successfully demonstrated *in vitro* using CHO cells as well as tumor cell lines (HT1080, MDA-MB-231, Supplementary Figure 3B). Washing the cells with Hanks’ Balanced Salt solution (HBSS) buffer 3 times prior to antibody incubation (∼933 nM) inhibited NHS76 internalization compared to unwashed cells, as did supplementation of the incubation buffer with excess H1. Light trypsinization of cells prior to antibody incubation greatly reduced NHS76 entry (data not shown). Trypsin hydrolyses proteins at their lysine or arginine residues. Histones are lysine and arginine-rich and thus are likely to be susceptible to trypsin proteolysis. Translocation of NHS76 into cells (Figure 6A) combined with the lack of any signal from a polyclonal mix of human IgG_1_/lambda antibodies (Figure 6B), indicates internalization is incumbent upon antibody specificity, that is the binding to histone or histone/DNA on the cellular surface, and not due to antibody interactions with alternate targets on the plasma membrane. To quantitate the relative difference in uptake, we again chose adherent cell lines for the reading of fluorescent signal from the internalized Rh110-TML labelled proteins (Supplementary Figure 3B).

Tumors were generated on chick embryo CAMs by topically applying a 1:1 mixture of MDA-MB-435 stable transfectants expressing mCherry-H1.2 or GFP. Tumors were grown until areas of necrosis were clearly visible, and then antibody was intravenously injected 24 hours prior to harvesting, fixing, embedding in OCT and sectioning for microscopy. Extensive distribution of NHS76 was observed in regions of necrotic nucleohistone debris as well as among adjacent viable tumor cells (Figures 6C and 6D). To localize NHS76 internalization in surrounding cells, immunolocalization was investigated either using two layers of secondary antibodies to amplify the signal (rabbit anti-human IgG followed by goat anti-rabbit conjugated to Alexa-750; Figures 6C and 6D) or using a goat anti-human secondary conjugated to quantum dots (655 nm) (Figure 6E). Injection of the negative control, tefibazumab, a humanized IgG_1_ targeting *Staphylococcus aureus* (Figure 6F and 6G), did not reveal significant localization in tumors. Even the vasculature, which was stained by injecting the lectin *Lens culinaris agglutinin*-A (*LCA*) 30 minutes prior to harvesting of the tumors, followed by staining of the tissue sections with a secondary antibody, goat anti-LCA conjugated to Alexa-647, was bereft of control antibody (Figure 6F). As a further control, injection of PBS and incubation of tissue sections with secondary antibody alone did not reveal any significant cross-reaction with cells (Figure 6H). In summary, these data demonstrate that the anti-histone antibody NHS76 accumulates in necrotic tumor tissue and the adjacent viable tumor, and that histones internalized by viable tumor cells *in vivo* are targeted by this antibody after intravenous injection.

## DISCUSSION

The necrotic regions of a tumor represent the most difficult to treat by conventional approaches such as radiation or chemotherapy due to resistance mediated by chronic hypoxia and impaired blood flow. Necrosis is an important disease target due to the prevalence of necrotic cell death in growing solid tumors and its contribution to the tumor microenvironment once a tumor is >4 mm in diameter [23]. The slow clearance of necrotic debris from this environment [19] makes it a useful target for antibody directed delivery of conjugated drugs to regions of hypoxia, where selection pressures help create highly resistant tumor cells. Two decades ago, researchers demonstrated that antibody penetration into the necrotic core of large solid tumors is feasible [24]. This was later found to be particularly true when the antibody has a weaker affinity for its target [18]. Here we present evidence that targeting histones may provide an attractive approach for drug delivery into cells located in these most difficult to treat areas.

To fully evaluate this approach, we had to clarify whether H1 translocation requires ATP energy-dependent endocytosis [25] or an independent process with a unique mechanism of internalization. If H1 translocation were energy-independent, then these proteins, and any antibodies targeting them, could passively diffuse into surrounding cells, including those cells with depleted ATP levels that are already on the verge of dying. An energy-dependent histone translocation process is advantageous for targeting viable tumor cells, particularly ones thriving in the hypoxic core of a tumor and utilizing ATP for endosomal internalization. Endocytosis can be blocked at low temperatures [26], which was the case when comparing H1 translocation at 37°C to 4°C using H1 labeled either with Alexa-488 (Figure 2B) or Rh110-TML (Figure 3B). Visually, H1 internalization involved vesicle formation (Figure 2A) regardless if the cell line is tumorigenic (N87) or not (CHO). Lysotracker Red DND-99 colocalization confirmed the H1 vesicles are acidic endosomes (Figures 2C and 3C). These results appear to contradict data from the Loyter lab suggesting histones translocate into cells independent of endocytosis or pinocytosis, however, their data focused on each of the core histones and not histone H1 [6]. We do not discount their observations showing evidence of histone internalization even when cultured cells are incubated with colchicine or cytochalasin D (known to inhibit microtubule function), nocodazole (known to depolymerize microtubules), nystatin (known to disrupt caveoloe formation) or sucrose (known to disrupt clathrin formation), or even at 4°C [6]. In fact, the Loyter lab has demonstrated core histone translocation into lipid bilayers lacking any endosomal machinery [7] and the Kinnunen lab has done the same for linker histones [8]. Our observations suggest that H1 internalization is largely driven by energy-dependent endocytosis, however, we are open to the idea that an alternative translocation mechanism also operates, albeit at a much lower rate, in cell culture conditions, perhaps even *in vivo*, but that has not been proven.

The most abundant isoform of H1 was chosen for our tracking study. Not only is it one of two subtypes critical for the functioning of a mammalian cell [12], it also signals the liberation of cytochrome C from the mitochondria during apoptosis in response to a DNA double-strand break [2]. Unlike earlier studies tracking exogenous histones applied to a culture of cells, we wanted to track endogenous histone migration *in vivo* by fusing the mCherry protein to the N-terminus of H1.2. H1-fluorescent protein fusions do not appear to perturb chromatin function *in vivo* [27], however, the C-tail is critical to H1 function so mCherry was fused to the N-tail [28]. Co-transfection of the HSV1-tk “suicide” gene allowed for controlled destruction of labeled H1 cells and the ability to track their histone content to cells not expressing mCherry-H1.2. The creation of these cells also allowed us a backup plan in the event sufficient necrosis did not occur naturally in the tumor microenvironment to allow tracking of mCherry-H1.2 migration from necrotic to viable cells. Upon *in vitro* validation (Figure 4), stable transfectants expressing mCherry-H1.2 or mCherry protein alone (“mCherry-Empty”) were mixed with GFP expressing cells and implanted as a polyclonal colony on a living CAM. Evaluation of tumor masses showed no significant difference whether the tumors were monoclonal or polyclonal in origin suggesting no inhibition of cellular activity due to expression of the fluorescent proteins (Figures 5A-5D). The use of mCherry-Empty cells provided a critical control showing the mCherry protein was not found in viable GFP-expressing tumor cells or CAM cells either due to fixation artifacts or any endosomal translocation process (Figure 5F). In contrast, mCherry-H1.2 translocation is documented with epifluorescent and confocal microscopy, revealing a dispersion pattern for H1 that is not focused in any one direction (Figures 5G-5K). We refer to this area surrounding the necrotic cell as the histone “blast zone” where histones and any molecules attached to them (e.g. DNA or antibodies) may be co-transported into a viable cell. Transfer of histones from donor cells to surrounding CAM cells could not be ruled out, and is likely to occur, however, the majority of necrotic cells in our tumor sections were surrounded by other tumor cells.

Anti-histone antibodies have been available to researchers for decades, largely derived from auto-immune diseases expressed in rodents and humans. Given the origins of these reagents, they are not always well-characterized and may exhibit cross-reactivity. Here we used an anti-H1/DNA derived from a human library screening [29] and provided the necessary characterization to show its specificity for a histone epitope that is accentuated upon binding to DNA (Figures 1). Naturally occurring autoimmune antibodies tend to target only a few epitopes on H1 despite the possible diversity of structures one would expect to find given the random structure of the histone tails [30]. The monoclonal NHS76 targets one of these epitopes, hence, providing a good homologue for many naturally occurring auto-antibodies. The role of naturally occurring anti-nuclear antibodies, including anti-histone or anti-nucleosomal ones, in aging individuals has been the topic of speculation recently with the proposal put forth that these molecules help combat cancer at a time when the immune system may be less vigilant [31, 32]. Affinity to the epitope is in the mid-nanomolar to micromolar range (Supplementary Figure 1); an advantage for diffusion of antibodies into the tumor core and interstitial spaces, although a smaller fraction of histones on the cell surface may be bound by NHS76 due to its weaker interaction strength [18].

Prior to *in vivo* testing, we verified NHS76 internalization *in vitro* using Rh110-TML labeled molecules (Figure 6A). Lack of signal using a polyclonal mix of human IgG_1_ / lambda indicates NHS76 internalization is not due to a general antibody receptor on the cell surface (Figure 6B). Excess H1 added to the incubation buffer inhibited translocation, as did light trypsinization of cells prior to NHS76 addition (data not shown). These results strongly suggest histones are a mediator of NHS76 internalization. The ability of H1 histones to mediate translocation of other proteins (e.g. thyroglobulin in cultured J774 macrophages [3]) and a variety of nucleic acids (e.g. [33]) has already been demonstrated in cell culture. The ability of histones to translocate anti-nucleosomal antibodies has also been demonstrated in culture [34]. Anti-nucleosomes were increasingly localized in cytoplasmic vesicles over the course of an 18 hour study, similar to what we saw with H1 (Figures 2). Whether this can occur *in vivo* is an open question. Readers versed in the autoimmune literature may think translocation of these types of antibodies has been well proven, however this work has largely been done in cell culture (e.g. [35]) while papers purporting to observe antibody translocations *in vivo* all the way into the nucleus turned out to be fixation artifacts [34]. A single electron micrograph illustrating vesicular translocation of a natural anti-nucleosomal antibody into mouse kidney cells is all that we are aware of as *in vivo* proof (Figure 5 in [34]). That study involved injection of anti-nucleosome producing hybridomas into mice to simulate an autoimmune condition. We have shown that anti-histones not only bind extracellular chromatin in regions of tumor necrosis (Figure 6C), they bind to viable cells (Figure 6D) and internalize (Figure 6E). The implications of anti-histone internalization *in vivo* are far reaching. This phenomenon may be responsible for the lower cancer mortality rates noted in some autoimmune patients [36, 37]. Aging individuals can have elevated anti-nucleosomal antibodies without signs of overt disease; with some of these antibodies able to selectively bind tumors rather than normal cells (e.g. MoAb 2C5 [31]). This has led to speculation that these non-pathogenic antibodies help compensate for other deteriorating immune responses to tumors in the aged [32]. While some suggest inducing autoimmune disease may help a patient fight cancer [38], we prefer using specific antibodies, such as NHS76, for intracellular delivery of conjugated drugs.

The implications of histone internalization *in vivo* are just as far reaching. While we have verified its occurrence in the tumor microenvironment, we know little about the physiological effects of histone release on viable tumor cells in the blast zone. If observations regarding another nuclear protein, HMGB1/amphoterin, are any guide, effects would not be implausible. During necrosis, HMGB1 diffuses out of necrotic cells and is known to interact with at least one cell membrane receptor, known as RAGE, as well as function as a cytokine mediator of inflammation (reviewed in [39]). Ample evidence of histones as agents of cell signaling and innate immunity [1] compels us to investigate similar roles for these proteins in the tumor microenvironment. Finally, it should be noted that some researchers use recombinant H1s to improve nucleic acid transfections of cultured cells (e.g. [33]). We have provided *in vivo* evidence of histone translocations into viable cells while others have provided evidence of histone/DNA (nucleosomal) internalization *in vitro* [10], however, the question remains for further investigation whether there are any physiological effects from the co-migration of DNA into viable tumor cells, particularly if that DNA contains genes responsible for further tumorigenesis.

## MATERIALS AND METHODS

### Reagents

Crystal violet (Cat. # 61135), Triton X-100 (Cat. # X100), Ganciclovir (Cat. # G2536), DAPI (Cat. # D9542), Hoechst 33258 (Cat. # B1155), DMF (*N, N*-Dimethylformamide; Cat. # 319937), DMSO (Dimethyl sulfoxide; Cat. # 472301), the cell culture media and buffers were purchased from Sigma-Aldrich. Chromatographically purified human IgG_1_ lambda (Cat. # 02-7102) was purchased from Zymed. Bovine H1 (from thymus; Cat. # 11 004 875 001) and serum albumin (Fraction V, protease free; Cat. # 03 117 332 001) were purchased from Roche. All other H1s were purified by Dr. Missag Parseghian. NHS76, a fully human IgG1, was produced at Peregrine Pharmaceuticals, Inc. (Tustin, CA). Tefibazumab, tradename Aurexis, was a kind gift from Inhibitex. All proteins were stored at 2-8°C until use. Alexa-Fluor 488 succinimidyl ester (Cat. # A20100) and Alexa-Fluor 594 C5 maleimide (Cat. # A10256), Lysotracker Red DND-99 (Cat. # L7528), Cell Tracker Blue CMHC (4-Chloromethyl-7-Hydroxycoumarin; Cat. # C2111) and trypsin (TrypLE™ Express, Cat. # 12604) were all purchased from Invitrogen. Calcein AM (Cat. # C3099) was from Life Technologies. Traut's reagent (2-iminothiolane) was purchased from Thermo Scientific (Cat. # 26101). SPDP (N-Succinimidyl 3-(2-pyridyldithio)-propionate) was purchased from Thermo Scientific (Cat. # 21857). Maleimidourea-rhodamine-110-trimethyl lock (Rh110-TML) was purchased from Dr. Ronald Raines (Univ. of Wisconsin).

### *In vitro* internalization studies and microscopy

For the *in vitro* studies, Chinese Hamster Ovary (CHO) fibroblast cells were grown in Ham's F-12 media with 10% fetal bovine serum (FBS). N87 cells were grown in RPMI with 10% FBS. HT1080, MDA-MB-231 and MDA-MB-435 cells were grown in DMEM with 10% FBS.

Conjugation of Alexa Fluor-488 to H1 using a succinimidyl ester occurred per manufacturer's instructions (Invitrogen Cat. # A20100). CHO fibroblast cells or N87 gastric carcinoma cells (ATCC, Manassas, VA) were grown on 8-well culture slides (Corning Cat. # 354108) incubated in a humidified 5% carbon dioxide (CO_2_) atmosphere set at 37°C. Wells were seeded with ∼35,000 cells suspended in 0.2 mL media. Upon adherence, cells were incubated with 5 μg/mL H1-Alexa Fluor-488 in media at 37°C for 0, 10, 30, 180 or 360 minutes. Before imaging, cells were rinsed with HBSS and incubated for 1.5 hours at 37°C and 5% CO_2_ with DAPI in HBSS at a high enough concentration (10 μg/ml) to allow for sufficient accumulation of DAPI within intact cells and diffusion to the nucleus. The cells were then incubated with buffer containing 0.25 mg/mL crystal violet and 0.001% Triton X-100 to quench any extracellular fluorescence prior to imaging.

Those cells stained with Lysotracker Red DND-99 were treated as above, however, they were incubated with 10 μg/ml H1-Alexa Fluor-488 in media for 17 hours at 37°C in a 5% CO_2_ incubator. Cell were then incubated with 50 nM Lysotracker Red DND-99 (Invitrogen Cat. # L7528) and 100 μg/ml Hoechst 33258 for 30 minutes at 37°C in a 5% CO_2_ incubator. The cells were then incubated with buffer containing 0.25 mg/mL crystal violet and 0.001% Triton X-100 to quench any extracellular fluorescence prior to imaging.

To investigate if H1 internalization is an energy-dependent process, CHO cells were plated at ∼35,000/well in 8-well culture slides and incubated in a 5% CO_2_ atmosphere set at 37°C until the cells adhered to the slide. Cells were then incubated with 10 μg/mL H1-Alexa Fluor-488 in media either at 37°C or on ice (4°C) for 3 hours in a 5% CO_2_ incubator. Cells were quickly removed from the incubator, rinsed with HBSS, and photographed.

Conjugation of Rh110-TMLto H1, BSA, NHS76 or Human IgG_1_ used the maleimide linkage integral to the molecule. Briefly, proteins were reduced under nitrogen with a 10-fold molar excess of 2 mg/mL 2-iminothiolane (2-IT) dissolved in dimethylformamide (DMF) for 2 hours before having the reaction stopped with a 10-fold molar excess of glycine for every mole of 2-IT. Samples were desalted on a Sephadex G-25 10/300 column with a flow rate of 2 mL/min using PBS buffer. The sulfhydryl-modified proteins were eluted and resuspended in a 5% DMF solution prior to incubation with a 10-fold molar excess of Rh110-TML (10 mg/mL in DMF) under nitrogen at 4°C for at least 16 hours. Samples were spun at 21,000 g for 5 minutes to remove insoluble material. Samples were then concentrated to about 1 mL in Ultraspin 15R centrifugal concentrators (Millipore). Concentrated proteins were buffer exchanged into a storage solution of 150 mM NaCl, 10 mM acetate (pH 5) and kept at 4°C until use.

Studies involving Rh110-TML labeled molecules (Figure 3B, 3C, 6A and 6B) also had cells plated into 8-well culture slides at ∼35,000/well and incubated at 37°C in a 5% CO_2_ incubator until they adhered. Cells were then incubated with 10 μg/ml of the Rh110-TML labelled protein in media at 37°C for the studies in Figures 3 and 6, except the temperature dependence study where some cells were incubated on ice (4°C) as described above. Incubations with DAPI (10 μg/ml) and Lysotracker Red DND-99 (50 mM) were done as described above. Only internalized molecules release a signal from the latent fluorophore, therefore, no quenching with crystal violet and 0.001% Triton X-100 was done for the experiments using Rh110-TML labelled proteins.

Studies to evaluate whether H1 mediates antibody internalization also required cells (∼35,000/well) to be plated into 8-well culture slides and incubated at 37°C in a 5% CO_2_ incubator until they adhered. For the wash studies, cells were rinsed 3X with 400 μL of HBSS and replaced with 200 μL of fresh media containing 210 μg/ml of NHS76-Rh110-TML or Human IgG_1_-Rh110-TML (∼933 nM). These cells, along with ones that were not washed prior to antibody incubation, were photographed 6 hours later and compared to see if the washed cells had less signal compared to the unwashed cells. For the H1 competition study, exogenous H1 and DNA, at a 1:5 ratio, was supplemented to the media and the cells plated into wells at ∼70,000/well. After overnight incubation at 37°C in a 5% CO_2_ incubator allowed for adherence, cells were incubated with 300 μL of fresh media containing 140 μg/ml of NHS76-Rh110-TML or Human IgG_1_-Rh110-TML (∼933 mM) for 6 hours at 37°C and 5% CO_2_. Cells were then imaged to see if excess H1 competed with the antigens targeted on the cell surface by NHS76-Rh110-TML. Finally, for the trypsinization study, ∼35,000 cells/well were plated into 8-well culture slides and incubated at 37°C in a 5% CO_2_ incubator until they adhered. Cells were then rinsed 3X with 400 μL of HBSS, incubated with trypsin (TrypLE™ Express, Invitrogen) for 2 minutes, rinsed 3X with 400 μL of HBSS again, and incubated for 3 hours with 200 μL of fresh media containing 210 μg/ml of NHS76-Rh110-TML or Human IgG_1_-Rh110-TML (∼933 nM). After 3 hours, the cells were washed 3X with 400 μL of HBSS to remove traces of antibody that had not internalized and then incubated for another 3 hours in 200 μL of fresh media prior to imaging to determine if trypsinization inhibited NHS76-Rh110-TML uptake into the cells.

Quantitation of cellular uptake for the Rh110-TML labelled molecules, involved having cells cultured in 96-well plates at ∼13,000/well and incubated at 37°C in a 5% CO_2_ incubator until they adhered. For Supplementary Figure 3A, cells were next incubated with a 1:2 serial dilution in cell culture media of Rh110-TML-H1.2 or Rh110-TML-BSA starting at a concentration of 10 μg/ml down to 156 ng/mL, in triplicate. For Supplementary Figure 3B, cells were incubated with a 1:2 serial dilution in cell culture media of NHS76-Rh110-TML or Human IgG_1_-Rh110-TML starting at a concentration of 150 μg/ml down to 2.3 μg/ml, in triplicate. Each well had a total final volume of 150 μL. Cells were incubated at 37°C in a 5% CO_2_ incubator overnight and then rinsed 2X with HBSS prior to reading in a spectrophotometer using excitation, emission and cut-off wavelengths of Ex = 464 nm, Em = 530 nm, cut-off = 475 nm.

Pictures were captured using a Reflected Fluorescence System with a CKX41 inverted microscope, oil immersion objective lens (100x) (all from Olympus), and a temperature controlled digital CCD camera mounted on top (ORCA-100 from Hamamatsu). The same exposure (75-100 msec) was used for all DAPI stained cells and exposure times were also kept consistent for Alexa Fluor-488 (100 msec) or Rh110-TML (800 msec) and Lysotracker Red (400 msec) photographs. Images were stored in a 16-bit grayscale format and then analyzed, colored and merged using Image J.

### Construction of cell Lines and *in vitro* migration study

HT1080, MDA-MB-231, and MDA-MB-435 cell lines were transfected with plasmids either bearing Green Fluorescent Protein (GFP), mCherry or mCherry-H1.2 using Lipofectamine 2000 (Invitrogen Cat. # 11668) per manufacturer's instructions. Plasmids were constructed with well established recombinant techniques (42). Vectors included a pUC origin for amplification in *E. coli* (DH5α) and an SV40 origin for replication in mammalian cells. The plasmids also included a CMV promoter 5′ to the mCherry protein, the mCherry-H1.2 fusion and the GFP. Plasmids were also created carrying the HSV1-tk coding sequence and its own CMV promoter 3′ of the fluorescent proteins and their H1 fusion sequences. Selection was conducted using a gene for neomycin resistance.

For the study in Figure 4, 1) HT1080 cells transiently transfected and expressing mCherry-H1.2 and HSV1-tk were cultured until they reached an exponential growth phase. 2) HT1080 cells transiently transfected and expressing mCherry-H1.2 without HSV1-tk were used as controls. 3) Untransfected HT1080s were grown in culture and then rinsed 3X with HBSS prior to incubation with CMHC (Invitrogen) in serum-free media at 37°C in a 5% CO_2_ incubator. After 30 minutes, the CMHC stained cells were rinsed 3X with serum-free media and then incubated in phenol red-free media at 37°C and 5% CO_2_ until their use in the experiment. Visual inspection with a microscope found those cells stained with 100 μM CMHC were bright enough for our study. Mixtures of CMHC stained “target” and mCherry-H1.2 and HSV1-tk expressing “donor” cells (15,000/well) were plated on to 8-well culture slides in two ratios, 50% donor : 50% target or 90% donor : 10 % target. To this 250 μL solution was added 50 μL of 300 μM ganciclovir, for a final concentration of 50 μM in 300 μL. Cells were allowed to adhere and grow in phenol red-free media overnight and then observed by microscopy. Mixtures of CMHC stained “target” and mCherry-H1.2 expressing “donor” cells without HSV1-tk were also cultured in the same manner as experimental controls. Another set of controls were also run, both sets of mixtures were cultured in the absence of ganciclovir with 50 μL more of phenol red-free media being added instead of ganciclovir to bring the final volume of media in each well to 300 μL.

### *In vivo* migration and internalization studies and microscopy

Chick embryos were extracted from their shells and kept in sterile plastic trays in a 37°C incubator *ex ovo* until tumor implantation as previously described (22). MDA-MB-435 cells expressing mCherry-H1.2 or mCherry-Empty were mixed 1:1 with MDA-MB-435 cells expressing GFP. Two million cells were topically implanted on the CAM of day 10 chick embryos and visualized directly using fluorescence microscopy.

For the H1 migration studies in Figure 5, polyclonal or monoclonal tumor colonies were grown on groups of 10 embryos each. Once the tumors appeared to be highly necrotic (day 18), CAMs from each group were divided into two sub-groups. Five of the embryos from each group were harvested, fixed in formaldehyde for 12 hours, followed by 70% ethanol and processed for hematoxylin and eosin staining (data not shown). For the remaining five embryos, the CAM was intravenously injected with a PBS solution containing CPMV labeled with Alexa-750 for detection of the chicken vasculature and incubated for 30 minutes at 37°C (41). The tumors were then harvested, fixed for 2 hours in a PBS solution containing 4% formalin, 10% sucrose and then embedded in OCT for 24 hours at -80°C (43). Tumors were then prepared for slides by sectioning (10 μm).

For the antibody migration studies in Figure 6, on day 17, twenty embryos each had 5 μg of NHS76 or tefibazumab intravenously injected into the CAM circulatory system in a volume of 50 μL. On day 18, tumor bearing CAMs from each group were split into two sub-groups. Ten of the embryos from each group were prepared for hematoxylin and eosin staining as described above (data not shown). The remaining ten embryos from each group were injected with a PBS solution containing 12.5 μg of unconjugated *Lens culinaris agglutinin*-A (*LCA*) for detection of the chicken vasculature and incubated for 30 minutes at 37°C. The tumors were then harvested, fixed for 2 hours in a PBS solution containing 4% formalin, 10% sucrose and then embedded in OCT for 24 hours at -80°C (43). Tumors were then prepared for slides by sectioning (10 μm). Sections stained with a secondary antibody, goat anti-LCA conjugated to Alexa-647 at 1 μg/mL, detected the vasculature. NHS76 or tefibazumab was detected by staining the tissue sections with a 2-step approach of rabbit anti-human IgG (5 μg/mL) followed by goat anti-rabbit conjugated to Alexa-750 (1 μg/mL). Finally, some tissue sections were also subjected to direct staining with NHS76 or tefibazumab, followed by the secondary antibodies. This was done to verify that the antigenic targets for NHS76 were present in the tissues and that the tefibazumab did not crossreact with other targets either specifically or non-specifically. These were controls that were done in the laboratory to verify the accuracy of our results, and while the results were as expected, the data is not presented in this study. The images seen in this paper are strictly NHS76 or tefibazumab that was intravenously injected before tissue harvesting and sectioning.

Non-tumorigenic cells were visualized, along with tumorigenic ones, using DAPI for detection of chromatin in the nuclei of all cells by staining tissue sections with a solution of ProLong^®^ Gold antifade with DAPI (Life Technologies Cat. # P36931).

Imaging of the tumor sections was performed using an upright epifluorescence microscope (AxioImager Z1, Carl Zeiss, Thornwood, NY) controlled by Volocity software (Improvision, Lexington, MA). Each fluorochrome was imaged against both negative and positive controls to establish optimal signal detection. Images for different sections were captured using similar exposure times for the different fluorochromes; images for different sections were similarly contrasted.

### Specificity studies

H1 and core histones were extracted from animal tissues as described by Parseghian et al. (1993)(44), except for bovine H1 (purchased from Roche) and the individual bovine core histones: H2A, H2B, H3 and H4 (purchased from Sigma-Aldrich). Creation of the H1° deletion mutants used for epitope localization are the kind gift of Dr. Jeffrey Hansen (Colorado State) and are described elsewhere (45).

For Figure 1A, whole cell extracts were derived from Raji cells (ATCC, Manassas, VA), fractionated by SDS-PAGE and transferred to nitrocellulose membranes for western blotting using procedures described in Parseghian et al. (1993) (44). Briefly, the extracts were created by suspending a volume of concentrated Raji cells in an equal volume of 2X SDS-PAGE loading dye and boiling the sample at 95°C for 5-10 minutes. Four wells were loaded on the SDS-PAGE for the analysis in Figure 1A with increasing volumes of 1.25 μL, 2.5 μL, 5 μL and 10 μL. To verify the proper loading and transfer of proteins to the nitrocellulose membranes, the protein bands were visualized using a general protein stain (BLOT-Fast Stain) according to the manufacturer's instructions (G Biosciences, Maryland Heights, MO). Western analysis occurred as described in Gao et al. (2004) (46), with a 3.6 μg/mL concentration of NHS76 used as the primary antibody and a 1:5000 dilution of goat anti-human IgG (Heavy & Light Chain) conjugated to alkaline phosphatase (Jackson Immunoresearch) as a secondary antibody.

Histones, their subtypes and the H1° fragments were resolved by SDS polyacrylamide gel electrophoresis (PAGE) and then blotted onto nitrocellulose using procedures described previously (44). For the gels in Figure 1B, 1μg of H1 was loaded from each species and 4 μg of core histones (containing ∼1 μg from each family). For Figure 1C, 0.5 μg of each H1 subtype was loaded per well. For Figure 1D, 1μg of each H1° deletion mutant was loaded per well. To verify the proper loading and transfer of proteins to the nitrocellulose membranes, the protein bands were visualized using BLOT-Fast Stain according to the manufacturer's instructions (G Biosciences). Western staining, blot washes and analysis occurred as described in Gao et al. (2004) (46), with the exception of the secondary antibodies. For Figure 1B and 1C, the antibodies were detected with a goat anti-human IgG (Heavy & Light Chain) conjugated to alkaline phosphatase (Jackson Immunoresearch) at a 1:5000 dilution. For Figure 1D, NHS76 was detected with a goat anti-human IgG (Heavy & Light Chain) conjugated to horseradish peroxidase (Pierce) at 1:5000.

For the ELISAs in Figure 1E characterizing the binding of antibody to histones and DNA, 0.5 μg of histones were placed into all wells, while those wells containing histones and DNA had 2.5 μg of DNA. In nature, the weight to weight ratio of histones:DNA in the nucleus is generally 1:1 (47); therefore, each of the five histone families has a ratio of only 1:5, which is what the 0.5 μg: 2.5 μg ratio was designed to mimic. Histones and DNA were crosslinked to withstand the stringent wash buffer used in these experiments. A crosslinking solution (11% formaldehyde, 0.1 M NaCl, 1 mM EDTA in 50 mM MES, pH 6.1) was added to the histone/DNA wells for a final concentration of 1% formaldehyde and then incubated for 8 minutes at room temperature before rinsing with water. All wells were then blocked (2% bovine serum albumin, 10 mM Tris-HCl, 150 mM NaCl, pH 7.5, 0.1% Micro-O-Protect) for 30 min, then incubated with 3 μg/mL of TNT antibody in blocking buffer for 1.5 hr at 37°C, before being washed (4% fish gelatin, 0.05% Tween 20, 10 mM Tris-HCl, 150 mM NaCl, pH 7.5) 3X for 5 min each. The antibodies were detected with a goat anti-human IgG (H & L Chain) conjugated to alkaline phosphatase at a 1:5000 dilution by incubating for 1 hr at 37°C, washing with fish gelatin 3X for 5 min each, rinsing with 10 mM Tris-HCl, 150 mM NaCl, pH 7.5 for 5 min, and then incubating with p-nitrophenyl phosphate substrate (Sigma Fast pNPP tablets, Sigma-Aldrich). Colorimetric analysis of the substrate cleavage was detected on a plate reader at 405 nm. Negative controls consisted of wells coated with each of the histone and histone/DNA antigens which were then probed only with the goat anti-human IgG conjugated to alkaline phosphatase secondary antibody at a 1:5000 dilution. The background values obtained from the control wells were subtracted from their respective wells that were probed with the primary antibody.

### Potency binding assay

Relative binding potency was determined in an ELISA format using a dose-response assay previously described in section 2.9 of Luhrs et al. (2009)(48). For the NHS76 inactivation study in Figure 1F, there was a modification to the secondary antibody step where the wells were incubated with 4 μg/mL goat anti-human IgG (H & L Chain) conjugated to Alexa Fluor 594 (Molecular Probes/Invitrogen) for 2 hours and then washed and read fluorometrically in a SpectraMax M5 (Molecular Devices) plate reader (*Ex* = 590 nm, *Em* = 625 nm, *Em Cutoff* = 610). To inactivate the NHS76, the antibody was placed in an aluminum foil wrapped tube and denatured by incubating it at 90°C for 15 minutes in Tris Buffered Salinie (TBS) before being cooled on ice.

### Immunohistochemistry

The HeLa cells used in Figure 1G were obtained from ATCC (Manassas, VA) and grown on poly-L-lysine coated coverslips. Cells were fixed with 4% para-formaldehyde/PBS for 10 minutes at room temperature, permeabilized for 10 minutes with 0.1% Triton X-100/PBS, then blocked with 10% Fetal Bovine Serum/PBS for 30 minutes. The cells were then stained with 5 μg/mL of NHS76. The washing and incubation followed well established procedures described elsewhere (49). The Alexa 594 conjugated goat anti-human IgG secondary antibody (1:200 dilution), Alexa 488 conjugated phalloidin (1:200 dilution) and DAPI (10 μg/mL) were all used according to the manufacturer's instructions (all from Molecular Probes/Invitrogen). Individual images were taken for each fluorochrome and then merged using ImageJ software from the NIH.

### Label-free binding studies

Binding was observed using biolayer interferometry (17), a label-free method that measures nanometer shifts in the interference of light that is reflected from the tip of an optical fiber probe (“a biosensor”) as the optical thickness changes with the association and dissociation of molecular interactions. Here we used an 8 biosensor system, the Octet (forté BIO, Menlo Park, CA). Each probe had a proprietary coating of streptavidin and all 8 were immersed into wells containing buffer designed to keep the cruciform DNA stable (10 mM MgCl_2_, 50 mM NaCl, 10 mM Tris-HCl, pH 7.5) (15). The cruciform structure was created from synthetic DNA fragments first described in Figure 1 of Bianchi (1988) (15) with the exception that one of our fragments bore a biotin at the 5′ end.

## SUPPLEMENTARY MATERIAL FIGURES



## Abbreviations

BSA: bovine serum albumin
C-terminal: carboxy terminal
CAM: chorioallantoic membrane
CHO: Chinese hamster ovary
CMHC: 4-chloromethyl-7-hydroxycoumarin
DAPI: 4′,6-diamidino-2-phenylindole
ELISA: enzyme linked immunosorbent assay
GFP: green fluorescent protein
HMGB: high mobility group B
HSV: herpes simplex virus
IgG: immunoglobulin
LCA: *Lens culinaris agglutinin*-A
MoAb: monoclonal antibody
NS: nucleosome
PS: phosphatidyl serine
Rh110-TML: rhodamine-110-trimethyl lock
SDS-PAGE: sodium dodecyl sulfate-polyacrylamide gel electrophoresis
TBS: tris buffered saline
tk: thymidine kinase

## References

[1] Parseghian MH, Luhrs KA. (2006). Beyond the walls of the nucleus: The role of histones in cellular signaling and innate immunity. Biochem Cell Biol.

[2] Konishi A, Shimizu S, Hirota J, Takao T, Fan Y, Matsuoka Y, Zhang L, Yoneda Y, Fujii Y, Skoultchi AI, Tsujimoto Y (2003). Involvement of histone H1.2 in apoptosis induced by DNA double-strand breaks. Cell.

[3] Brix K, Summa W, Lottspeich F, Herzog V (1998). Extracellularly occurring histone H1 mediates the binding of thyroglobulin to the cell surface of mouse macrophages. J Clin Invest.

[4] Evans DL, Kaur H, Leary III J, Praveen K, Jaso-Friedmann L (2005). Molecular characterization of a novel pattern recognition protein from nonspecific cytotoxic cells: Sequence analysis, phylogenetic comparisons and anti-microbial activity of a recombinant homologue. Dev Comp Immunol.

[5] Lundberg M, Johansson M (2002). Positively charged DNA-binding proteins cause apparent cell membrane translocation. Biochem Biophys Res Commun.

[6] Hariton-Gazal E, Rosenbluh J, Graessmann A, Gilon C, Loyter A Direct translocation of histone molecules across cell membranes. J Cell Sci.

[7] Rosenbluh J, Hariton-Gazal E, Dagan A, Rottem S, Graessmann A, Loyter A (2005). Translocation of histone proteins across lipid bilayers and Mycoplasma membranes. J Mol Biol.

[8] Zhao H, Bose S, Tuominen EKJ, Kinnunen PKJ (2004). Interactions of histone H1 with phospholipids and comparison of its binding to giant liposomes and human leukemic T cells. Biochemistry.

[9] Iakoubov LZ, Torchilin VP (1998). Nucleosome-releasing treatment makes surviving tumor cells better targets for nucleosome-specific anticancer antibodies. Cancer Detect Prev.

[10] Koutouzov S, Cabrespines A, Amoura Z, Chabre H, Lotton C, Bach JF (1996). Binding of nucleosomes to a cell surface receptor: redistribution and endocytosis in the presence of lupus antibodies. Eur J Immunol.

[11] Parseghian MH, Hamkalo BA (2001). A compendium of the histone H1 family of somatic subtypes: An elusive cast of characters and their characteristics. Biochem Cell Biol.

[12] Parseghian MH (2015). What is the role of histone H1 heterogeneity? A functional model emerges from a 50 year mystery. AIMS Biophysics.

[13] Parseghian MH, Henschen AH, Krieglstein KG, Hamkalo BA (1994). A proposal for a coherent mammalian histone H1 nomenclature correlated with amino acid sequences. Protein Sci.

[14] Zlatanova JS, Srebreva LN, Banchev TB, Tasheva BT, Tsanev RG (1990). Cytoplasmic pool of histone H1 in mammalian cells. J Cell Sci.

[15] Bianchi ME (1988). Interaction of a protein from rat liver nuclei with cruciform DNA. EMBO J.

[16] Varga-Weisz P, Zlatanova JS, Leuba SH, Schroth GP, van Holde KE (1994). Binding of histones H1 and H5 and their globular domains to four-way junction DNA. Proc Natl Acad Sci USA.

[17] Concepcion J, Witte K, Wartchow C, Choo S, Yao D, Persson H, Wei J, Li P, Heidecker B, Ma W, Varma R, Zhao LS, Perillat D (2009). Label-free detection of biomolecular interactions using BioLayer interferometry for kinetic characterization. Comb Chem High Throughput Screen.

[18] Thurber GM, Schmidt MM, Wittrup KD (2008). Factors determining antibody distribution in tumors. Trends Pharmacol Sci.

[19] Napirei M, Wulf S, Mannherz HG (2004). Chromatin breakdown during necrosis by serum DNAse1 and the plasminogen system. Arthritis Rheum.

[20] Lavis LD, Chao TY, Raines RT (2006). Fluorogenic label for biomolecular imaging. ACS Chem Biol.

[21] Shaner NC, Campbell RE, Steinbach PA, Giepmans BN, Palmer AE, Tsien RY (2004). Improved monomeric red, orange and yellow fluorescent proteins derived from Discosoma sp. red fluorescent protein. Nat Biotechnol.

[22] Pink DBS, Schulte W, Parseghian MH, Zijlstra A, Lewis JD (2012). Real-time visualization and quantitation of vascular permeability in vivo: Implications for drug delivery. PLoS ONE.

[23] Li X-F, Carlin S, Urano M, Russell J, Ling CC, O’Donoghue JA (2007). Visualization of hypoxia in microscopic tumors by immunofluorescent microscopy. Cancer Res.

[24] Chen F-M, Epstein AL, Li Z, Taylor CR (1990). A comparative autoradiographic study demonstrating differential intratumor localization of monoclonal antibodies to cell surface (Lym-1) and intracellular (TNT-1) antigens. J Nucl Med.

[25] Schmid SL, Carter LL (1990). ATP is required for receptor-mediated endocytosis in intact cells. J Cell Biol.

[26] Kuismanen E, Saraste J (1989). Low temperature-induced transport blocks as tools to manipulate membrane traffic. Methods Cell Biol.

[27] Gunjan A, Alexander BT, Sittman DB, Brown DT (1999). Effects of H1 histone variant overexpression on chromatin structure. J Biol Chem.

[28] Th’ng JPH, Sung R, Ye M, Hendzel MJ (2005). H1 family histones in the nucleus: Control of binding and localization by the C-terminal domain. J Biol Chem.

[29] Sharifi J, Khawli LA, Hu P, King S, Epstein AL (2001). Characterization of a phage display-derived human monoclonal antibody (NHS76) counterpart to chimeric TNT-1 directed against necrotic regions of solid tumors. Hybrid Hybridomics.

[30] Stemmer C, Briand JP, Muller S (1994). Mapping of linear epitopes of human histone H1 recognized by rabbit anti-H1/H5 antisera and antibodies from autoimmune patients. Mol Immunol.

[31] Iakoubov L, Rokhlin O, Torchilin V (1995). Anti-nuclear autoantibodies of the aged reactive against the surface of tumor but not normal cells. Immunol Lett.

[32] Torchilin VP, Iakoubov LZ, Estrov Z (2001). Antinuclear autoantibodies as potential antineoplastic agents. Trends Immunol.

[33] Puebla I, Esseghir S, Mortlock A, Brown A, Crisanti A, Low W (2003). A recombinant H1 histone-based system for efficient delivery of nucleic acids. J Biotechnol.

[34] Kramers K, van Bruggen MC, Rijke-Schilder TP, Dijkman HB, Hylkema MN, Croes HJ, Fransen JA, Assmann KJ, Tax WJ, Smeenk RJ, Berden JH (1996). In vivo ANA is a fixation artifact: nucleosome-complexed antinucleosome autoantibodies bind to the cell surface and are internalized. J Am Soc Nephrol.

[35] Golan TD, Gharavi AE, Elkon KB (1993). Penetration of autoantibodies into living epithelial cells. J Invest Dermatol.

[36] Palo J, Duchesne J, Wikstrom J (1977). Malignant diseases among patients with multiple sclerosis. J Neurol.

[37] Sadovnick AD, Eisen K, Ebers GC, Paty DW (1991). Cause of death in patients attending multiple sclerosis clinics. Neurology,.

[38] Pardoll DM (1999). Inducing autoimmune disease to treat cancer. Proc Natl Acad Sci USA.

[39] Bianchi ME, Manfredi A (2004). Chromatin and cell death. Biochim Biophys Acta.

[40] Albig W, Meergans T, Doenecke D (1997). Characterization of the H1.5 gene completes the set of human H1 subtype genes. Gene.

[41] Leong HS, Steinmetz NF, Ablack A, Destito G, Zijlstra A, Stuhlmann H, Manchester M, Lewis JD (2010). Intravital imaging of embryonic and tumor neovasculature using viral nanoparticles. Nat Protoc.

[42] Maniatis T, Fritsch EF, Molecular Sambrook J (1982). Cloning: A Laboratory Manual.

[43] Kusser KL, Randall TD (2003). Simultaneous detection of EGFP and cell surface markers by fluorescence microscopy in lymphoid tissues. J Histochem Cytochem.

[44] Parseghian MH, Clark RF, Hauser LJ, Dvorkin N, Harris DA, Hamkalo BA (1993). Fractionation of human H1 subtypes and characterization of a subtype-specific antibody exhibiting non-uniform nuclear staining. Chromosome Res.

[45] Lu X, Hamkalo BA, Parseghian MH, Hansen JC (2009). Chromatin condensing functions of the linker histone C-terminal domain are mediated by specific amino acid composition and intrinsic protein disorder. Biochemistry.

[46] Gao S, Chung YG, Parseghian MH, King GJ, Adashi EY, Latham KE (2004). Rapid H1 linker histone transitions following fertilization or somatic cell nuclear transfer: evidence for a uniform developmental program in mice. Dev Biol.

[47] van Holde KE (1988). Chromatin.

[48] Luhrs KA, Harris DA, Summers S, Parseghian MH (2009). Evicting hitchhiker antigens from purified antibodies. J Chromatogr B.

[49] Harlow E, Lane D (1988). Antibodies: A Laboratory Manual.

