# Supplementary material for: In vivo histone H1 migration from necrotic to viable tissue
Source: Oncotarget. 2017 Feb 7;8(10):16275–92. doi: 10.18632/oncotarget.15181 (PMC5369962; doi:10.18632/oncotarget.15181)
Supplement: Supplementary file 1 [file oncotarget-08-16275-s001.pdf]

In vivo histone H1 migration from necrotic to viable tissue

Supplementary Material

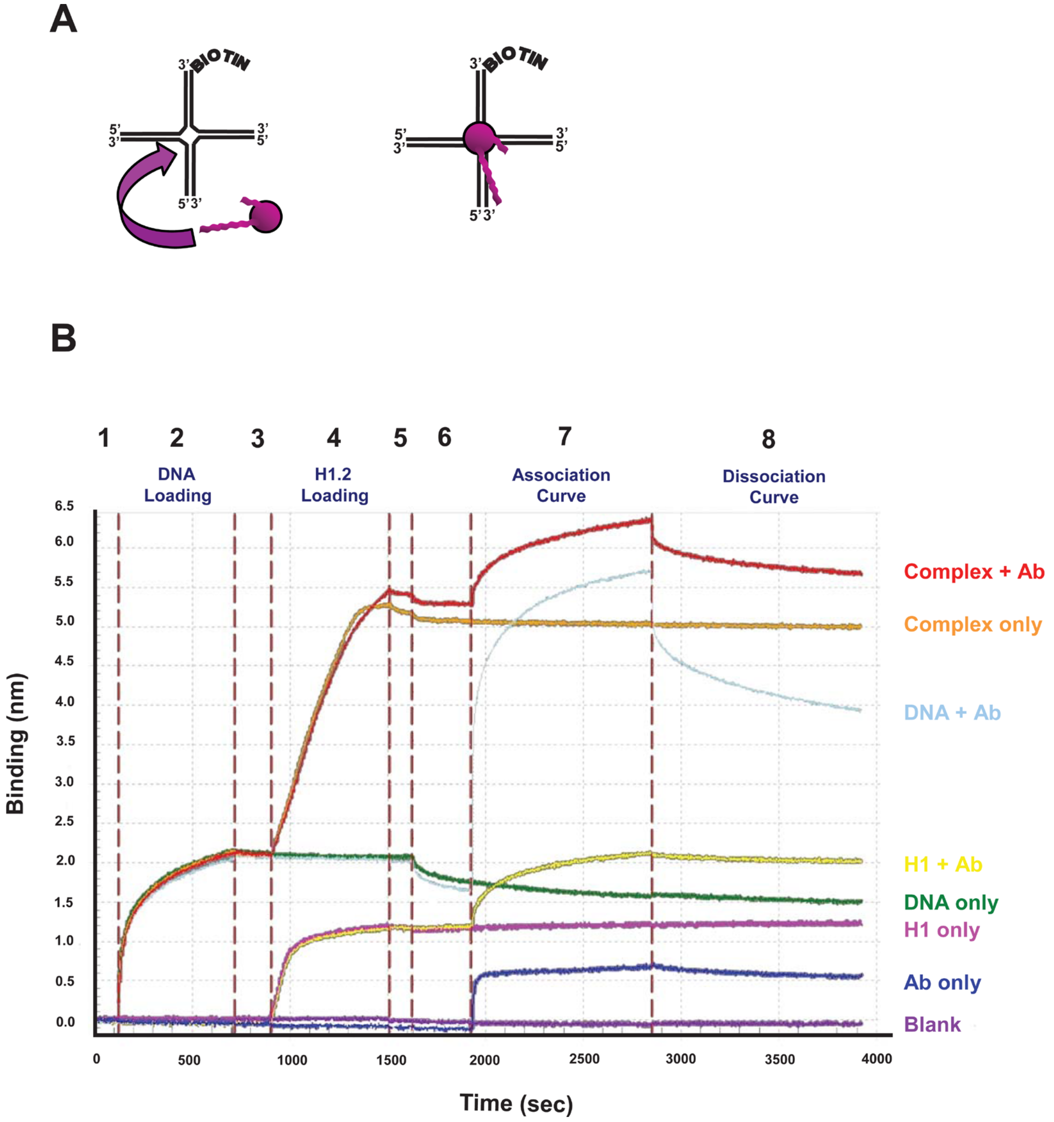

Supplementary Figure 1. NHS76 binding studies for H1/DNA complex. (A) Schematic representation of a defined antigenic complex consisting of a single H1 molecule interacting at the junction of a 4-way DNA cruciform structure. One strand of DNA was biotinylated to allow for the cruciform structure's binding to the streptavidin coated probe. (B) Summary of steps taken to observe binding with biolayer interferometry. For our initial survey, a baseline measurement was obtained with all eight probes in a modified form of a buffer designed to keep the cruciform DNA stable (10 mM $MgCl_2$, 50 mM NaCl, 10 mM Tris-HCl, pH 7.5) [1] (15). Four probes were then immersed in 15 µg/mL biotinylated cruciform DNA and their binding with the streptavidin coating recorded on the y-axis as a nanometer scale phase shift in the reflected wavelength of light [2] (red, orange, green and light blue traces). The probes were rinsed in buffer [3] followed by an immersion of two DNA containing probes in a 100 nM solution of H1.2 for creation of the H1/DNA complex [4] (16) (red and orange traces). Two other probes lacking DNA were also immersed in a solution of 100 nM biotinylated H1.2 [4] (yellow and purple traces). A final rinse in buffer resulted in pairs of probes with H1/DNA complex (red and orange traces), as well as H1 alone (yellow and purple traces), DNA alone (green and light blue traces) and no antigen at all (dark blue and violet traces) [5]. The probes were then immersed in phosphate buffered saline (PBS) to match the antibody buffer [6] before being exposed to the 1 µM antibody (Ab) solution [7]. Upon having the association curve achieve a signal plateau, the probes were immersed in PBS buffer lacking any antibody and dissociation from the probes was recorded [8]. Probes immersed in 1 µM antibody (Ab) solution are represented with red, light blue, yellow and dark blue traces. Corresponding probes not immersed in antibody solution are represented with orange, green, purple and violet traces.

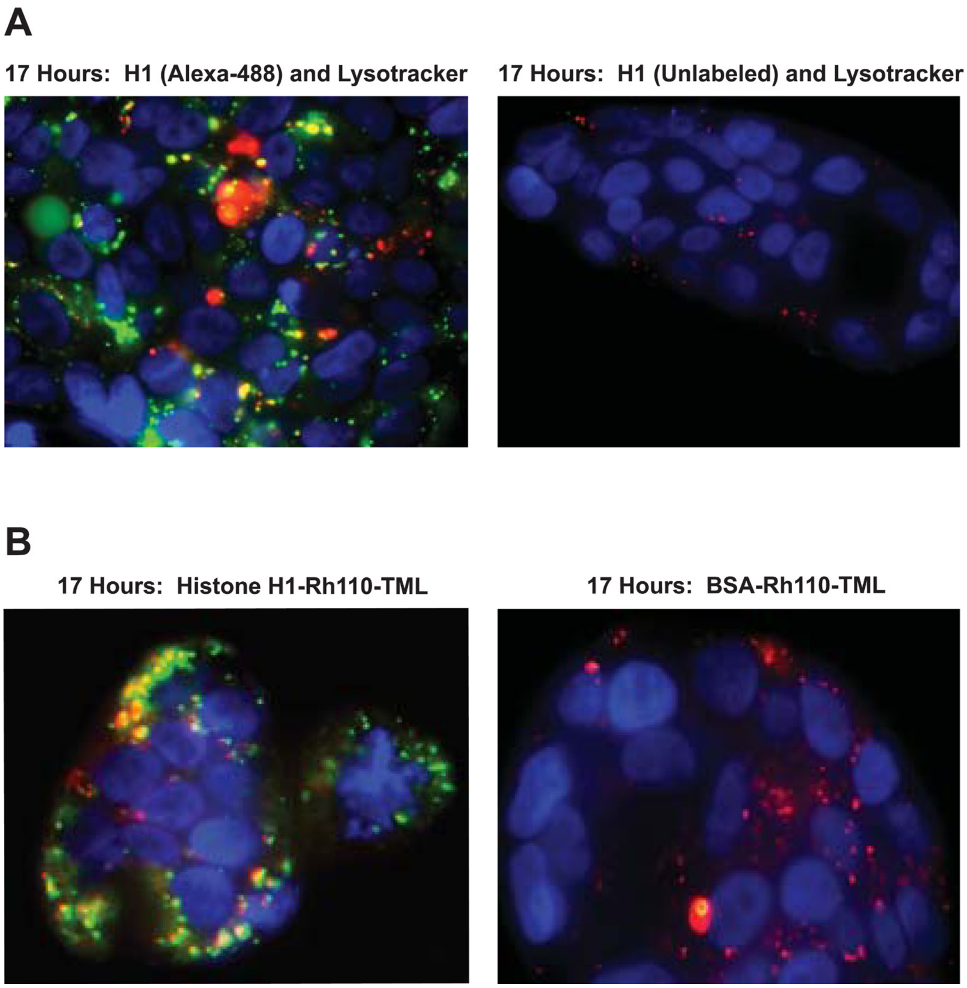

Supplementary Figure 2. Live Cell Imaging of H1 Uptake in N87 gastric carcinoma cells. (A) Colocalization of histone H1 in the acidic endosomal compartment seen in non-tumorigenic CHO cells was also seen with N87 carcinoma cells. Cells incubated with Alexa-488 or unlabeled H1 (10 µg/ml) at 37°C for 17 hours were further stained, during the last 30 minutes prior to microscopy, with 50 nM Lysotracker Red and 100 µg/ml Hoechst 33258, a cell permeable DNA stain. (B) Rh110-TML-labeled BSA or histone H1 (both 10 µg/ml) were incubated with N87 cells for 17 h at 37°C. During the final 30 min, LysoTracker Red and Hoechst 33258 were added as in (A). Images were taken of live, unfixed cells. Exposure times and image processing were identical for each image.

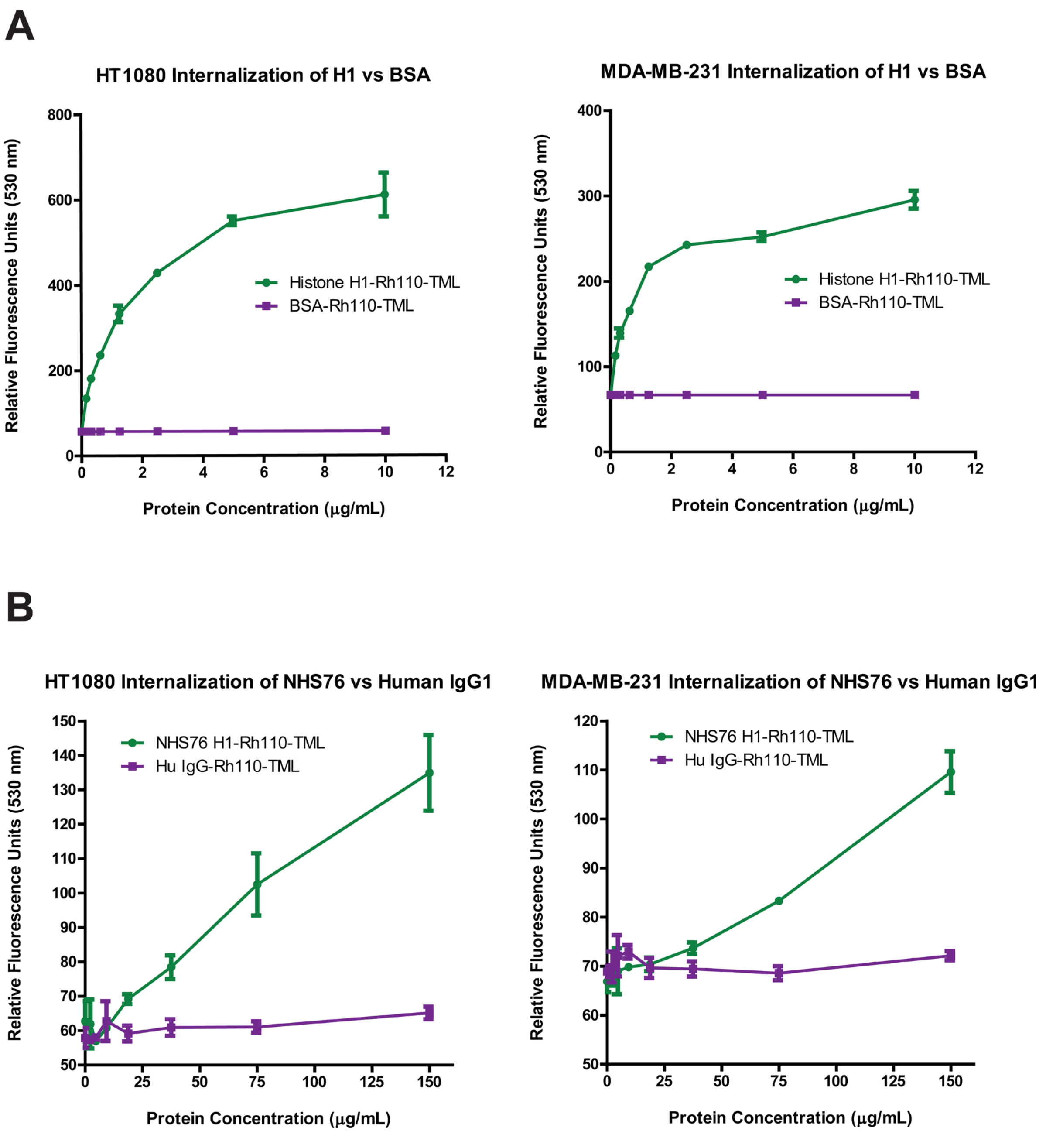

Supplementary Figure 3. Quantitation of cellular uptake for the Rh110-labelled molecules. (A) HT1080 fibrosarcomas and MDA-MB-231 adenocarcinomas were plated into 96-well plates (~13,000 cells/well) and incubated at 37°C in a 5% CO_2 incubator until they adhered. Cells were then incubated with a 1:2 serial dilution in cell culture media of Histone H1-Rh110-TML or BSA-Rh110-TML starting at a concentration of 10 µg/ml down to 156 ng/mL, in triplicate. After overnight incubation at 37°C in a 5% CO_2 incubator, wells were rinsed twice with HBSS buffer and then read in a spectrophotometer at 530 nm. (B) Cells were similarly incubated with a 1:2 serial dilution in cell culture media of NHS76-Rh110-TML or Human IgG_1-Rh110-TML starting at a concentration of 150 µg/ml down to 2.3 µg/ml, in triplicate. After overnight incubation at 37°C in a 5% CO_2 incubator, wells were rinsed twice with HBSS buffer and then read in a spectrophotometer at 530 nm.
